# Food and Microbiota Metabolites Associate with Cognitive Decline in Older Subjects: A 12‐Year Prospective Study

**DOI:** 10.1002/mnfr.202100606

**Published:** 2021-10-28

**Authors:** Raúl González‐Domínguez, Pol Castellano‐Escuder, Francisco Carmona, Sophie Lefèvre‐Arbogast, Dorrain Y. Low, Andrea Du Preez, Silvie R. Ruigrok, Claudine Manach, Mireia Urpi‐Sarda, Aniko Korosi, Paul J. Lucassen, Ludwig Aigner, Mercè Pallàs, Sandrine Thuret, Cécilia Samieri, Alex Sánchez‐Pla, Cristina Andres‐Lacueva

**Affiliations:** ^1^ Biomarkers and Nutrimetabolomics Laboratory Faculty of Pharmacy and Food Sciences University of Barcelona Barcelona 08028 Spain; ^2^ CIBER Fragilidad y Envejecimiento Saludable (CIBERfes) Instituto de Salud Carlos III Madrid 28029 Spain; ^3^ Department of Genetics Microbiology and Statistics University of Barcelona Barcelona 08028 Spain; ^4^ University of Bordeaux Inserm Bordeaux Population Health Research Center UMR 1219 Bordeaux F‐33000 France; ^5^ Université Clermont Auvergne INRAE UNH Clermont Ferrand F‐63000 France; ^6^ Department of Basic and Clinical Neuroscience Maurice Wohl Clinical Neuroscience Institute Institute of Psychiatry Psychology and Neuroscience King's College London London SE5 9NU UK; ^7^ Brain Plasticity Group Swammerdam Institute for Life Sciences Center for Neuroscience University of Amsterdam Amsterdam 1098 XH The Netherlands; ^8^ Institute of Molecular Regenerative Medicine Spinal Cord Injury and Tissue Regeneration Center Salzburg Paracelsus Medical University Salzburg 5020 Austria; ^9^ Pharmacology Section Department of Pharmacology Toxicology and Medicinal Chemistry Faculty of Pharmacy and Food Sciences, and Institut de Neurociències University of Barcelona Barcelona 08028 Spain

**Keywords:** cognitive decline, diet, food metabolome, gut microbiota, metabolomics

## Abstract

**Scope:**

Diet is considered an important modulator of cognitive decline and dementia, but the available evidence is, however, still fragmented and often inconsistent.

**Methods and Results:**

The article studies the long‐term prospective Three‐City Cohort, which consists of two separate nested case‐control sample sets from different geographic regions (Bordeaux, n = 418; Dijon, n = 424). Cognitive decline is evaluated through five neuropsychological tests (Mini‐Mental State Examination, Benton Visual Retention Test, Isaac's Set Test, Trail‐Making Test part A, and Trail‐Making Test part B). The food‐related and microbiota‐derived circulating metabolome is studied in participants free of dementia at baseline, by subjecting serum samples to large‐scale quantitative metabolomics analysis. A protective association is found between metabolites derived from cocoa, coffee, mushrooms, red wine, the microbial metabolism of polyphenol‐rich foods, and cognitive decline, as well as a negative association with metabolites related to unhealthy dietary components, such as artificial sweeteners and alcohol.

**Conclusion:**

These results provide insight into the early metabolic events that are associated with the later risk to develop cognitive decline within the crosstalk between diet, gut microbiota and the endogenous metabolism, which can help identify potential targets for preventive and therapeutic strategies to preserve cognitive health.

## Graphical abstract

A large‐scale quantitative metabolomics platform is employed to obtain a deeper insight into the role of diet in the early onset of cognitive decline. Strong evidence supports a protective association between metabolites derived from cocoa, coffee, mushrooms, red wine, the microbial metabolism of polyphenol‐rich foods and cognitive decline. The article also finds a negative association with metabolites related to unhealthy dietary components, such as artificial sweeteners and alcohol.

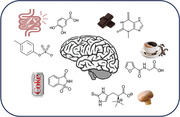

## 1 Introduction

The involvement of modifiable lifestyle factors in the etiology of age‐related cognitive decline (CD) and dementia is well recognized.^[^ ^1^ ^]^ In particular, nutrition has been identified as a pivotal player in maintaining proper brain function with increasing age.^[^ ^2^ ^]^ Indeed, many dietary components can modulate the molecular mechanisms that are thought to contribute to CD, including oxidative stress, neuroinflammation and vascular dysfunction.^[^ ^3^ ^]^ Several studies have even suggested a protective role of certain nutrients and food compounds (e.g., omega‐3 fatty acids, B vitamins, polyphenols, carotenoids), food groups (e.g., fruits and vegetables) and dietary patterns (e.g., Mediterranean diet, Dietary Approaches to Stop Hypertension diet) against CD.^[^ ^3^, ^4^ ^]^ However, most of this available evidence is observational and, often, inconsistent and fragmented.^[^ ^3^, ^5^ ^]^ Part of these inconsistencies may be due to misreporting errors inherent to the food intake surveys that are commonly employed for dietary assessment, whereas biomarker‐based studies, which are less prone to measurement errors, have been usually limited to only a few candidate biomarkers.^[^ ^5^ ^]^ As an alternative, metabolomics offers comprehensive assessment of the food and endogenous metabolome, thereby enabling the investigation of the impact of diet on health in great detail in a reliable manner.^[^ ^6^ ^]^ Therefore, the large‐scale investigation of food‐related metabolites is crucial in order to truly elucidate a role for lifelong nutrition in the early pathogenesis of CD. Several untargeted metabolomics studies have been published before that focused on the metabolic pathways and molecular mechanisms behind CD and dementia.^[^ ^7^, ^8^ ^]^ In this respect, we recently identified a set of serum metabolites associated with the subsequent development of CD over a 12‐year follow‐up, which comprised six diet‐derived metabolites that were related to the consumption of coffee, cocoa, citrus and other foods.^[^ ^9^ ^]^ However, although untargeted metabolomics enables wide metabolome coverage, it usually hinders the detection of minor metabolites, especially those coming from external sources (e.g., diet and other lifestyle habits) that are generally detected at low concentrations in the organism. As a complementary strategy, the use of large‐scale targeted platforms, which offer improved sensitivity, reproducibility and coverage, has emerged in recent years for quantitative metabolomics analysis, with great applicability in nutrimetabolomics^[^ ^10^, ^11^ ^]^ and exposomics research.^[^ ^12^ ^]^

**Figure 1:**
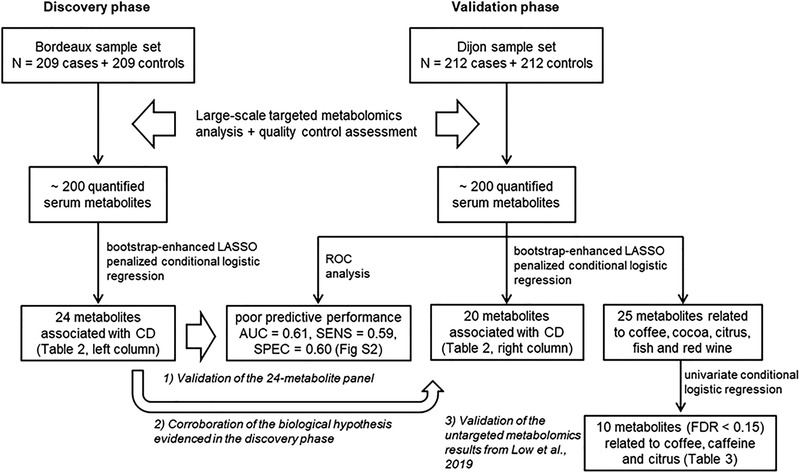
Schematic representation of the study design.

Therefore, the aim of this study was to decipher the role of diet in the pathogenesis of CD by applying a large‐scale targeted metabolomics approach, which encompasses polyphenolic and other food‐origin compounds, phase I/II metabolites, microbiota‐transformed derivatives and other endogenous metabolites.^[^ ^12^ ^]^ This advanced methodology was applied to serum samples from a large cohort of older subjects free of dementia at the time of blood draw from the Three‐City (3C) Cohort.^[^ ^13^ ^]^ For validation purposes, we investigated two separate long‐term prospective sample sets from different study centers (i.e., Bordeaux and Dijon). The metabolomics platform enabled us to obtain an in‐depth insight into the involvement of nutrition in CD, and to validate our previous untargeted metabolomics results in a separate population^[^ ^9^ ^]^ (see **Figure** **1** for a schematic representation of the study design).

## 2 Results

### 2.1 Characteristics of the Study Populations

Clinical and demographic characteristics of the study populations at baseline are shown in **Table** **1**. The participants were matched for age, gender and education level within the two samples, and these characteristics were similar between the discovery (i.e., Bordeaux) and validation (i.e., Dijon) sets. As expected, lower scores for the five neuropsychological tests assessed in this study were observed among CD participants. Medication intake was significantly higher within cases than controls, as well as the prevalence of diabetes and cardiovascular diseases, and the presence of the APOE‐ε4 allele, which are well‐known risk factors for CD and dementia.^[^ ^14^ ^]^

**Table 1:** Clinical and demographic characteristics of the discovery (Bordeaux) and validation (Dijon) case‐control samples

	Bordeaux sample set		Dijon sample set	
	Cases (N = 209)	Controls (N = 209)	Cases (N = 212)	Controls (N = 212)
Age (years)	75.9 ± 4.5	75.7 ± 4.2	76.5 ± 5.2	76.1 ± 4.7
Gender (male/female)	71/138	71/138	78/134	78/134
Education level, ≥ secondary school (%)	28.7	28.7	28.3	28.3
BMI (kg m^–2^)	26.8 ± 4.4	26.1 ± 3.6	25.8 ± 4.6	25.1 ± 3.6
Mini‐Mental State Examination	26.9 ± 2.2	28.0 ± 1.6	25.9 ± 2.4	28.7 ± 1.0
Benton Visual Retention Test	10.8 ± 2.1	11.7 ± 1.9	10.0 ± 2.3	12.6 ± 1.6
Isaac's Set Test	27.7 ± 5.9	31.0 ± 6.1	28.0 ± 6.6	38.0 ± 6.2
Trail‐Making Test part A	24.2 ± 7.9	29.4 ± 9.1	22.2 ± 9.6	35.6 ± 11.1
Trail‐Making Test part B	10.0 ± 5.8	14.5 ± 6.5	7.8 ± 5.0	19.0 ± 6.7
Number of medications regularly consumed	4.9 ± 2.7	4.1 ± 2.4	5.5 ± 3.0	4.0 ± 3.0
ApoE‐ε4 (%)	25.8	12.0	26.9	20.8
Diabetes (%)	12.9	5.7	12.7	5.7
History of cardiovascular diseases (%)	33.5	27.8	41.0	30.2

### 2.2 Identification of Serum Metabolites Associated with Subsequent Cognitive Decline

The Bordeaux metabolomics data matrix was subjected to bootstrap‐enhanced LASSO penalized conditional logistic regression to identify a set of metabolites associated with subsequent CD over the 12‐year follow‐up, as shown in **Table** **2** (the concentrations detected within each study group are listed in Table S1, Supporting Information). We observed an inverse association of various phenolic acids and derivatives with CD, which may be ingested from plant‐based foods (i.e., fruits and vegetables) and/or be produced through the microbial metabolism of plant polyphenols. However, a few other phenolic compounds linked to the metabolism of aromatic amino acids (e.g., 4‐HPAA‐G, DOPAC‐S) were associated with increased odds of CD. It should be noted that, although this second set of phenolic‐related compounds might also derive from plant polyphenols, Pearson's correlation analysis showed strong associations among them (r >0.30, Figure S1, Supporting Information) but not with most of the other phenolic metabolites listed in Table 2; thus, pointing to a different origin. This further supports our hypothesis that these metabolites could accurately mirror alterations in the metabolism of aromatic amino acids rather than in the intake of dietary polyphenols. Other metabolites identified by LASSO regression were candidate food intake biomarkers, as defined by the Food Biomarker Ontology.^[^ ^15^ ^]^ On the one hand, markers reflecting the consumption of cocoa (3‐methylxanthine, OR = 0.75), coffee (2‐furoylglycine, OR = 0.57) and mushrooms (ergothioneine, OR = 0.90) were associated with a reduced risk of CD. In contrast, serum levels of caffeine (OR = 1.88), artificial sweeteners (saccharin, OR = 1.26; acesulfame K, OR = 1.12), tartaric acid (OR = 1.36) and proline betaine (OR = 1.74) were higher among CD cases. Moreover, our results also provide evidence for significant alterations in some endogenous metabolic pathways, including tryptophan homeostasis (indoxyl sulfate, OR = 0.75), fatty acid metabolism (myristic acid, OR = 2.10; linoleoyl‐carnitine, OR = 3.67) and others (betaine, OR = 0.54; DHEAS, OR = 0.68).

**Table 2:** Metabolites associated with subsequent cognitive decline in both the discovery (Bordeaux) and validation (Dijon) samples identified by bootstrap‐enhanced least absolute shrinkage and selection operator (LASSO) penalized conditional logistic regression (metabolites with >40% selection across bootstraps in at least one of the samples are presented)

Metabolites	Bordeaux sample set		Dijon sample set	
	Frequency of selection across bootstraps	Odds ratio	Frequency of selection across bootstraps	Odds ratio
Phenolic acids and derivatives				
Vanillin	82.9	0.71		
3‐Hydroxybenzoic acid sulfate (3‐HBA‐S)	72.3	0.93	73.3	0.62
2‐Hydroxybenzoic acid (2‐HBA)	71.8	0.71		
3,4‐Dihydroxybenzoic acid (3,4‐DHBA)	61.3	0.88		
3‐Hydroxyphenylacetic acid sulfate (3‐HPAA‐S)	50.3	0.89	43.1	0.84
Dihydroferulic acid sulfate (DHFA‐S)	48.5	0.60		
3‐Hydroxyhippuric acid (3‐HHA)			56.5	0.71
Xanthine alkaloids				
3‐Methylxanthine	63.3	0.75	48.7	0.87
Caffeine	61.1	1.88		
1,3‐Dimethyluric acid			71.1	0.48
Artificial sweeteners				
Saccharin	69.7	1.26	43.2	1.34
Acesulfame K	45.7	1.12		
Other food‐related metabolites				
Umbelliferone sulfate	70.4	0.51		
2‐Furoylglycine	53.8	0.57		
Tartaric acid	52.8	1.36		
Proline betaine	44.4	1.74		
Enterolactone sulfate	43.5	0.65		
5‐(4”‐Hydroxy‐3”‐methoxyphenyl)‐γ‐valerolactone sulfate (MHPV‐S)	43.3	0.98	55.4	1.22
Ergothioneine	41.6	0.90	59.2	0.72
Ethyl sulfate			56.6	1.82
cis‐Resveratrol 3‐sulfate			41.3	0.70
Aromatic amino acid derivatives				
Indoxyl sulfate	48.0	0.75		
4‐Hydroxyphenylacetic acid glucuronide (4‐HPAA‐G)	49.2	1.43	54.1	1.25
3,4‐Dihydroxyphenylacetic acid sulfate (DOPAC‐S)	42.0	1.09		
4‐Hydroxyphenyllactic acid sulfate (4‐HPLA‐S)			75.0	1.50
5‐Hydroxytryptophan			69.1	0.41
4‐Methylcatechol sulfate (4‐MeCAT‐S)			55.5	1.43
Phenylacetylglutamine			49.8	2.33
p‐Cresol sulfate			49.1	1.40
Serotonin			48.1	0.66
Fatty acids and derivatives				
Myristic acid	58.5	2.10		
Lauric acid			63.5	2.08
Linoleoyl‐carnitine	52.6	3.67		
Other endogenous metabolites				
Betaine	43.3	0.54		
Dehydroepiandrosterone sulfate (DHEAS)	40.7	0.68		
Citric acid			41.8	0.79
Thiamine			43.8	1.64

### 2.3 Validation of the Metabolomics Results in an External Sample

To validate the metabolomics findings uncovered in the discovery phase, we first attempted to evaluate the predictive ability of the 24‐metabolite panel previously identified by LASSO regression as a biomarker to discriminate cases and controls in the external validation sample of Dijon. However, receiver operating characteristic analysis of this serum metabolite signature yielded poor area under the curve (AUC = 0.61), sensitivity (SENS = 0.59) and specificity (SPEC = 0.60) (Figure S2, Supporting Information). Next, as an alternative approach for external validation, the entire Dijon metabolomics dataset was subjected to LASSO regression for identifying the metabolites associated with CD in this separate sample set (Table 2, right column). Among the 20 metabolites associated with CD that were identified in the validation sample, seven were common to those found in the discovery phase, most of which showed the same direction of association with CD in both study samples (3‐HBA‐S, 3‐HPAA‐S, 3‐methylxanthine and ergothioneine were associated with decreased odds of CD, whereas 4‐HPAA‐G and saccharin were associated with increased odds of CD). Noteworthy, the LASSO results from the Dijon sample accurately validated some of the findings described in the discovery phase regarding the role of certain dietary compounds on CD, such as the inverse association with phenolic acids, 3‐methylxanthine (i.e., cocoa intake) and ergothioneine (i.e., mushroom intake), as well as the deleterious association with saccharin. In addition, this analysis also suggested a protective association of metabolites potentially reflecting red wine consumption (cis‐resveratrol 3‐sulfate, OR = 0.70), whereas ethyl sulfate, a marker of total alcohol intake, was associated with increased odds of subsequent CD (OR = 1.82). Nevertheless, contradictory results were found regarding the involvement of caffeine on the onset of CD, that is, an inverse association was found here in the validation sample between a caffeine metabolite (1,3‐dimethyluric acid, OR = 0.48) and CD, while a deleterious association was observed in the discovery sample (caffeine, OR = 1.88). Besides this diet‐CD interplay, the application of LASSO to the validation sample also demonstrated the pivotal involvement of aromatic amino acids and fatty acids in the early pathogenesis of CD, in agreement with the results unveiled in the discovery stage. Interestingly, similar results were obtained when applying LASSO‐penalized conditional logistic regression to the total sample (i.e., combination of the populations from Bordeaux and Dijon); thus, corroborating the robustness of these metabolite‐CD associations (Table S2, Supporting Information).

In a complementary approach, univariate conditional logistic regression was applied to the validation set to investigate case‐control differences in serum levels of metabolites related to the intake of the foods discovered in our previous untargeted metabolomics study.^[^ ^9^ ^]^ As shown in **Table** **3**, this corroborated the protective association of several coffee‐related metabolites (i.e., trigonelline, N‐methylpyridinium, cyclo(leucyl‐proline)) with CD. However, this secondary univariate analysis showed that circulating levels of various caffeine derivatives (i.e., caffeine, paraxanthine, 1‐methylxanthine, 1,3‐dimethyluric acid, 1‐methyluric acid) and citrus derived metabolites (i.e., proline betaine, 4‐hydroxyproline betaine) were also decreased among CD cases in the prospective sample of Dijon (Table 3), which was not consistent with the results observed in the Bordeaux sample using both targeted and untargeted metabolomics.

**Table 3:** Food‐related metabolites selected by univariate conditional logistic regression (FDR‐adjusted *p*‐values <0.15) in the validation sample

Metabolites	Odds ratio	FDR‐adjusted *p*‐value
1‐Methylxanthine	0.54	0.0150
1,3‐Dimethyluric acid	0.60	0.0150
Paraxanthine	0.49	0.0183
Caffeine	0.36	0.117
Trigonelline	0.40	0.124
*N*‐methylpyridinium	0.34	0.124
Cyclo(Leucyl‐Proline)	0.34	0.124
4‐Hydroxyproline betaine	0.33	0.124
1‐Methyluric acid	0.21	0.126
Proline betaine	0.31	0.126

## 3 Discussion

Numerous efforts have been made to elucidate the impact of nutrition on CD during aging, but the available evidence is often inconsistent and fragmented.^[^ ^3^, ^5^ ^]^ To investigate the role of diet in the etiology of CD from a more long‐term perspective, we studied here two nested case‐control prospective sample sets over a 12‐years follow‐up among older subjects free of dementia at baseline, from whom baseline serum samples were collected and subjected to large‐scale metabolomics analysis.

**Figure 2:**
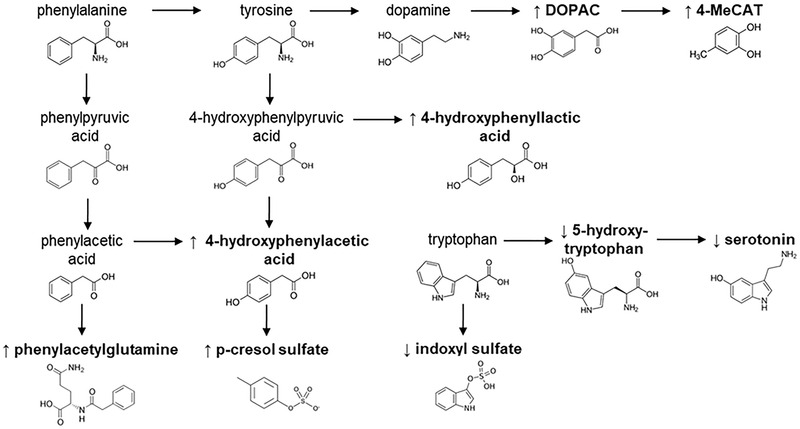
Overview of the CD‐related disturbances in the metabolism of aromatic amino acids. Metabolites associated with greater (↑) or lower (↓) odds of CD in at least one of the two study sample sets are marked in bold.

Many of the metabolites identified in the two study samples sets by LASSO regression, including polyphenol derivatives (e.g., phenolic acids, enterolignans, hydroxyphenyl‐γ‐valerolactones) and aromatic amino acid metabolites, suggest a close interplay between diet, gut microbiota and CD. We observed an inverse association between various phenolic acids and other plant‐related metabolites with the odds of subsequent CD, which provides additional evidence for a protective effect of polyphenol‐rich food consumption in neurological dysfunction.^[^ ^16^ ^]^ Noteworthy, most of these metabolites were microbial‐derived compounds (i.e., phenolic acids, enterolignans) rather than the parent polyphenol species. In this respect, mounting evidence has emphasized that microbiota metabolites could be involved, at least in part, in the biological effects that are traditionally attributed to polyphenols, especially in view of their usual low bioavailability.^[^ ^17^ ^]^ Indeed, microbiota‐derived phenolic acids have been linked to CD‐related processes, such as oxidative stress, neuroinflammation, accumulation of β‐amyloid plaques and hyper‐phosphorylation of tau protein, especially since these metabolites can be present in the circulation at significantly higher concentrations for longer periods of time, and can easily cross the blood‐brain barrier.^[^ ^18^, ^19^ ^]^ On the other hand, we also found significant associations between microbiota‐derived aromatic amino acid metabolites and subsequent CD (**Figure** **2**). Various metabolites related to the catabolism of phenylalanine and tyrosine were associated with greater odds of CD in the Bordeaux (i.e., 4‐HPAA‐G) and Dijon (i.e., 4‐HPAA‐G, 4‐HPLA‐S, phenylacetylglutamine, p‐cresol sulfate) samples, which is in agreement with previous studies reporting that these compounds may act as uremic toxins and cause cognitive impairment.^[^ ^20^, ^21^, ^22^, ^23^ ^]^ The positive association of 3,4‐dihydroxyphenylacetic acid sulfate (Bordeaux) and 4‐methylcatechol sulfate (Dijon), downstream metabolites of dopamine, with subsequent CD supports the involvement of disrupted monoaminergic neurotransmission in the early onset of dementia.^[^ ^24^ ^]^ In this respect, other metabolic disturbances affecting tryptophan homeostasis corroborated this monoaminergic dysfunction in CD, as suspected from the decreased levels of indoxyl sulfate, 5‐hydroxytryptophan and serotonin in serum samples from subjects with a higher CD rate during the follow‐up (Figure 2). Furthermore, this was accompanied by abnormal levels of thiamine, which is essential together with other B‐group vitamins for regulating the tryptophan metabolism, further supporting this argument.^[^ ^25^ ^]^

Besides the above mentioned perturbations in metabolites involved in the gut‐brain axis, we also found associations with various food intake biomarkers, which enabled us to investigate the potentially protective or deleterious effect of various food products on the rate of CD. In line with our previous untargeted metabolomics study,^[^ ^9^ ^]^ we here observed a negative association between 3‐methylxanthine, a metabolite derived from theobromine that is present in cocoa, and subsequent CD in the discovery ad validation sets (Table 2). Notably, 3‐methylxanthine serum levels were highly correlated with theobromine (r > 0.60), and although not selected by LASSO regression among the top metabolites with the highest frequency of selection across bootstraps, theobromine was also strongly and negatively associated with CD in both sample sets (OR = 0.52 and 0.55, respectively). All this, therefore, reinforces the beneficial effect of cocoa consumption against CD, which is in accordance with other prospective studies.^[^ ^26^ ^]^ 2‐Furoylglycine, a biomarker of coffee consumption,^[^ ^27^ ^]^ was associated with lower odds of CD in the discovery set. This metabolite was not successfully validated in the external study sample, but complementary univariate analysis showed reduced serum levels of various other coffee‐related metabolites (i.e., trigonelline, *N*‐methylpyridinium, cyclo(leucyl‐proline)) among CD cases (Table 3). This protective role of coffee on CD has been repeatedly reported in other observational studies,^[^ ^28^ ^]^ although the effect of caffeine itself is still under debate.^[^ ^29^ ^]^ In this respect, we found contradictory results in the discovery (caffeine, OR = 1.88) and validation (1,3‐dimethyluric acid, OR = 0.48) sample sets by LASSO regression, which was further corroborated by univariate statistical analysis (Table 3). This inconsistency could in part be due to the large person‐to‐person variability in the metabolism and sensitivity to caffeine,^[^ ^29^ ^]^ so future research is needed to better understand this coffee‐caffeine paradox. Besides cocoa and coffee related metabolites, we also observed a decreased content of serum ergothioneine in CD cases, which is a naturally occurring histidine derivative that is acquired through the diet, mainly from mushrooms.^[^ ^30^ ^]^ Previous studies have reported that blood levels of ergothioneine tend to decline with age in the elderly,^[^ ^31^, ^32^ ^]^ and its administration protects hippocampal neurons against oxidative stress.^[^ ^33^ ^]^ So far, it remains unclear whether this ergothioneine decrease during aging and the onset of CD is due to nutritional deficiencies, disturbances in its absorption, or oxidative modifications.^[^ ^32^ ^]^

In addition to the potential protective associations between polyphenol‐rich foods, cocoa, coffee, mushrooms and CD, our metabolomics data also pointed to a harmful association with certain dietary components, including artificial sweeteners and proline betaine (Table 2). Accumulating evidence with animal models suggests that chronic intake of non‐caloric artificial sweeteners may impair cognitive performance by causing dysregulation of the energy metabolism and oxidative stress.^[^ ^34^, ^35^ ^]^ However, this is, to our knowledge, the first time that this hypothesis linking artificial sweeteners with CD is corroborated from a long‐term perspective, and also validated in two separate sample sets. Proline betaine, a biomarker of citrus intake,^[^ ^36^ ^]^ was also positively associated with CD in the discovery phase, in line with our previous untargeted metabolomics study^[^ ^9^ ^]^ and others.^[^ ^37^ ^]^ Furthermore, this metabolite was correlated with serum levels of hesperitin 3’‐glucuronide (r = 0.44), a characteristic flavonoid of citrus fruits that was also strongly associated with greater odds of subsequent CD (OR = 2.90, but not selected by LASSO). In our untargeted metabolomics study, we suggested that this observation could be attributed to the consumption of commercial juices rather than raw fruits, considering that correlation analyses between metabolomics and food intake data showed a strong correlation between proline betaine and citrus juice intake, but not with citrus fruit intake.^[^ ^9^ ^]^ Conversely, univariate statistics revealed a protective association of citrus markers (i.e., proline betaine, 4‐hydroxyproline betaine) with CD in the validation set of Dijon (Table 3). Although not corroborated with food intake data (not available for the validation set), this is in line with previous studies reporting that the consumption of total citrus fruits is related to lower risk of dementia.^[^ ^38^ ^]^

Aside from the food and microbiota‐related compounds discussed above, the metabolomics signatures we identified also comprised other endogenous metabolites. A remarkable finding in this respect was the accumulation of fatty acids and acyl‐carnitines in CD subjects, which could be indicative of impaired β‐oxidation, as described in other metabolomics studies on CD and dementia.^[^ ^8^ ^]^ Moreover, the findings regarding betaine, DHEAS and citric acid could also reflect the involvement of other central metabolic pathways (e.g., one‐carbon metabolism, tricarboxylic acid cycle) in CD pathogenesis, in accordance with previous literature.^[^ ^8^ ^]^

The major strengths of our study include the application of a powerful metabolomics approach and the use of a population‐based prospective design. From an analytical point of view, our work provides the most comprehensive characterization of the human food metabolome performed up‐to‐date on CD research. This is due to the application of a large‐scale quantitative metabolomics platform comprising food‐related compounds, phase I/II metabolites, microbiota‐transformed derivatives as well as other metabolites involved in the endogenous metabolism. The application of this methodology to baseline serum samples from a long‐term prospective trial over a 12‐year follow‐up has enabled the investigation of etiological risk factors in a very early phase, prior to CD and the appearance of dementia symptoms. Furthermore, the use of two separate nested case‐control sample sets considerably increased the reliability of the hypotheses here generated. However, some limitations deserve to be mentioned as well. Only baseline serum samples were available for metabolomics analysis, and we thus could not examine longitudinal changes in the food metabolome during CD development. In this vein, it should also be noted that the 12‐year storage of samples from the 3C Study could have impacted the levels of some labile metabolites, although many studies have demonstrated that long‐term storage in ultra‐freezers does not considerably influence the metabolic composition of blood.^[^ ^39^ ^]^ Furthermore, although metabolites can be regarded as surrogate dietary biomarkers, they are also prone to errors (e.g., lack of specificity, inter‐individual variability factors), so the potential associations between foods and CD presented in this study must be interpreted with caution. Noteworthy, the validation of the multi‐metabolite panel discovered in the Bordeaux sample set yielded poor predictive performance in the external sample of Dijon (Figure S2, Supporting Information). Nevertheless, this was not especially surprising, as many authors have repeatedly reported inconsistent results and unsatisfactory validation of metabolomics‐based biomarkers.^[^ ^40^, ^41^, ^42^, ^43^ ^]^ Growing evidence points that one of the most important challenges for biomarker validation studies in the metabolomics field could be the high intra‐ and inter‐individual variability of the human metabolome, which can arise from genetic (e.g., gender), temporal (e.g., circadian rhythm), environmental (e.g., dietary habits) or microbial (e.g., eubiosis/dysbiosis) factors.^[^ ^44^ ^]^ This variability may result in different, but analogous, metabolic responses (e.g., different metabolites from the same metabolic pathway) to a given physiological or pathological stimuli. For this reason, our study aim was to validate the biological hypotheses generated in the discovery stage, not individual metabolites, using the external validation set.

## 4 Concluding Remarks

In conclusion, our prospective and validated data suggest that food‐related and microbiota‐derived metabolites may play an important role in the later development of CD. Our results support a protective association between metabolites reflecting the consumption of polyphenol‐rich foods (i.e., fruits and vegetables), cocoa, coffee, mushrooms and red wine with CD, whereas other food components related to unhealthy dietary components (i.e., alcohol, artificial sweeteners) may have deleterious effects on cognition. In this respect, the apparent paradoxes regarding the binomials coffee‐caffeine and red wine‐total alcohol deserve further research. Moreover, we found evidence for disturbances in central metabolic pathways, such as the microbiota‐modulated metabolism of aromatic amino acids, β‐oxidation of fatty acids, and others. Finally, our study highlights the great impact of inter‐individual variation on metabolomics and, consequently, on the adequate performance of external validation studies. Interestingly, although many of the metabolites that were identified were different between the two study samples, these revealed consistent associations of certain food intake patterns and central metabolic pathways with CD. This reinforces not only the added value of validating global biological/metabolic pathways rather than single metabolites, but also stresses the need for moving beyond biomarkers towards mechanisms and pathways in metabolomics research.

## 5 Experimental Section

### Study Design

Two nested case‐control sample sets were built among participants from the centers of Bordeaux and Dijon of the Three‐City (3C) Cohort, with Bordeaux as the discovery set and Dijon for external validation. The 3C study is a population‐based cohort on dementia that includes older persons (>65 years) from three French cities (Bordeaux, Dijon and Montpellier).^[^ ^13^ ^]^ Sociodemographic and lifestyle characteristics, medical information, neuropsychological testing, blood pressure, anthropometric measurements and fasting serum samples were collected at baseline, and follow‐up visits were then scheduled every 2–3 years for neuropsychological assessment. All blood samples were collected in fasting conditions to minimize the influence of food intake before blood drawing and variations associated with the circadian rhythm. The study was performed in accordance with the principles contained in the Declaration of Helsinki. The Consultative Committee for the Protection of Persons participating in Biomedical Research at Kremlin‐Bicêtre University Hospital (Paris, France) approved the 3C study protocol, and all participants provided written consent.

From the entire Bordeaux and Dijon cohorts, eligible participants were selected for the present study if they were not diagnosed with dementia at baseline, had available serum samples, and had at least one repeated cognitive evaluation over the subsequent 12 years. To build the case‐control samples on CD, a composite score of global cognition was defined at each follow‐up visit as the average of *Z*‐scores of five neuropsychological tests (Mini‐Mental State Examination, Benton Visual Retention Test, Isaac's Set Test, Trail‐Making Test part A, and Trail‐Making Test part B).^[^ ^45^, ^46^, ^47^, ^48^ ^]^ Individual slopes of cognitive change were then evaluated using linear mixed models, as detailed in the previous publication.^[^ ^9^ ^]^ Cases were defined as the participants with the worst slopes of CD (209 cases in Bordeaux and 212 cases in Dijon). Then, each case was matched to a control (i.e., a participant with a slope of CD better than the population median) with the same age, gender and education level.

### Metabolomics Analysis of Serum Samples

Metabolomics analysis of serum samples was conducted using a large‐scale and quantitative multi‐metabolite platform for the simultaneous detection and quantification of food‐related metabolites, microbiota derivatives and endogenous metabolites, following the methodology developed by González‐Domínguez et al.^[^ ^12^ ^]^ First, serum samples were subjected to protein precipitation by mixing 100 µL of each study sample with 250 µL of methanol containing 0.1% formic acid.^[^ ^9^ ^]^ The samples were vigorously vortexed for 30 s, and centrifuged at 10 000  × *g* for 10 min at 4 °C. The supernatant (250 µL) was collected, mixed with 250 µL acetonitrile and transferred to 96‐well injection plates. A set of internal standards was added to the samples for quantification and quality control assessment. The samples were randomly distributed for metabolomics analysis by ultra‐high performance liquid chromatography coupled to tandem mass spectrometry using the operating conditions described elsewhere.^[^ ^12^ ^]^

### Statistical Analysis

Metabolomics data were first pre‐processed using a standardized protocol developed in‐house. Missing values were imputed using the KNN algorithm, and data were then log‐transformed and Pareto‐scaled. Afterwards, Euclidean distances to the group centroid were computed to remove outlier samples from the data matrix (±3×IQR). The coefficients of variation for peak areas, retention times and peak widths of the internal standards were computed for evaluating the analytical reproducibility. After this pre‐processing, 206 metabolites passed quality control and were considered for further statistical analysis.

In the discovery phase (Bordeaux sample), Least Absolute Shrinkage and Selection Operator (LASSO) penalized conditional logistic regression was employed to select the metabolites that were significantly associated with the odds of subsequent CD, according to the previously described methodology.^[^ ^9^ ^]^ The model was conditioned on matching variables (i.e., age, gender and education level) and adjusted for the non‐penalized variables, body mass index (BMI) and total number of medications regularly consumed. Furthermore, bootstrap resampling (i.e., 1000 bootstrapped samples) was applied to guide the variable selection, as LASSO regression may lead to unstable solutions.^[^ ^49^ ^]^ Accordingly, the metabolites selected by LASSO in at least 40% of the bootstrapped samples were considered as the variables reliably associated with CD. Then, unpenalized conditional logistic regression was used to estimate the unbiased multivariable adjusted odds ratio (OR) for each selected metabolite (confidence intervals were not estimated as known to be biased in post‐selection inference).^[^ ^50^ ^]^

For validation purposes, the Area Under the Receiver Operating Characteristic Curve was first computed to evaluate the ability of the multi‐metabolite panel built in the discovery sample (i.e., Bordeaux) by means of unpenalized conditional logistic regression for predicting the odds of CD in the external validation set (i.e., Dijon). As an alternative validation approach, instead of validating individual metabolites, LASSO regression was applied to the entire Dijon metabolomics data matrix to identify the metabolites associated with CD in this external validation sample with the aim of corroborating the overall biological hypotheses evidenced in the discovery phase. To check the strength of the associations between serum metabolites and CD, the total sample (i.e., combining the populations from Bordeaux and Dijon) was also subjected to LASSO‐penalized conditional logistic regression, conditioned on matching variables and adjusted for BMI and medication intake.

Furthermore, the article also aimed to validate in a separate study population (i.e., Dijon) the associations discovered in the previous untargeted metabolomics study between metabolites derived from coffee, cocoa, citrus, fish and red wine, and subsequent CD in the Bordeaux sample set.^[^ ^9^ ^]^ To this end, the article pre‐selected from the Dijon dataset those metabolites quantified through the metabolomics platform that may reflect the consumption of these food items, as described in the *Food Biomarker Ontology*:^[^ ^15^ ^]^ cocoa (i.e., theobromine, 3‐methylxanthine, cyclo(prolyl‐valine)), coffee (i.e., 2‐furoylglycine, trigonelline, *N*‐methylpyridinium, cyclo(leucyl‐proline)), caffeinated food products (i.e., caffeine, 1‐methylxanthine, paraxanthine, 1‐methyluric acid, 1,3‐dimethyluric acid), citrus (i.e., proline betaine, 4‐hydroxyproline betaine, naringenin 7‐glucuronide, hesperetin 3’‐glucuronide, hesperetin 7‐glucuronide, hesperetin 7‐sulfate), red wine (i.e., cis‐resveratrol 3‐sulfate, trans‐resveratrol 3‐sulfate, ethyl sulfate) and fish (i.e., 3‐methylhistidine, trimethylamine N‐oxide, eicosapentaenoic acid, docosahexaenoic acid). This set of 25 food intake biomarkers was then subjected to false discovery rate (FDR)‐corrected univariate conditional logistic regression, which was conditioned on the matching variables and adjusted for BMI and the total number of medications regularly consumed. Metabolites with FDR‐corrected *p*‐values below 0.15 were selected for further discussion.

## Authors' Contributions

Conceptualization, R.G.‐D., C.M., A.K., P.J.L., L.A., S.T., C.S., C.A.‐L.; Data curation, R.G.‐D., P.C.‐E., F.C., A.S.‐P.; Formal Analysis, R.G.‐D., P.C.‐E., F.C., S.L.‐A., A.S.‐P.; Funding acquisition, C.M., A.K., P.J.L., L.A., M.P., S.T., C.S., C.A.‐L.; Investigation, R.G.‐D.; Methodology, R.G.‐D., P.C.‐E., F.C., S.L.‐A., C.S., A.S.‐P.; Project administration, C.M., A.K., P.J.L., L.A., M.P., S.T., C.S., C.A.‐L.; Resources, C.S., A.S.‐P., C.A.‐L.; Software, P.C.‐E., F.C., A.S.‐P.; Supervision, R.G.‐D., A.S.‐P., C.A.‐L.; Validation, R.G.‐D., P.C.‐E., F.C., A.S.‐P.; Visualization, R.G.‐D.; Writing – original draft, R.G.‐D.; Writing – review & editing, R.G.‐D., P.C.‐E., F.C., S.L.‐A., D.Y.L., A.D.P., S.R.R., C.M., M.U.‐S., A.K., P.J.L., L.A., M.P., S.T., C.S., A.S.‐P., C.A.‐L. All authors read and approved the final manuscript.

## Conflict of Interest

The authors declare no conflict of interest.

## Supporting information

Supporting Information

## Data Availability

The data used in the current study are available from the corresponding author on reasonable request.
