# Supplementary material for: Food and Microbiota Metabolites Associate with Cognitive Decline in Older Subjects: A 12‐Year Prospective Study
Source: Mol Nutr Food Res. 2021 Oct 28;65(23):2100606. doi: 10.1002/mnfr.202100606 (PMC11475530; doi:10.1002/mnfr.202100606)
Supplement: Supplementary file 1 — Supporting Information [file MNFR-65-2100606-s001.pdf]

Food and microbiota metabolites associate with cognitive decline in older subjects: A twelve-year prospective study

Raúl González-Domínguez[1,2*], Pol Castellano-Escuder[1,2,3], Francisco Carmona[2,3], Sophie Lefèvre-Arbogast[4], Dorrain Y. Low[5], Andrea Du Preez[6], Silvie R. Ruigrok[7], Claudine Manach[5], Mireia Urpi-Sarda[1,2], Aniko Korosi[7], Paul J. Lucassen[7], Ludwig Aigner[8], Mercè Pallàs[9], Sandrine Thuret[6], Cécilia Samieri[4], Alex Sánchez-Pla[2,3], Cristina Andres-Lacueva[1,2*]

[1]Biomarkers and Nutrimetabolomics Laboratory, Faculty of Pharmacy and Food Sciences, University of Barcelona, 08028 Barcelona, Spain. [2]CIBER Fragilidad y Envejecimiento Saludable (CIBERfes), Instituto de Salud Carlos III, 28029 Madrid, Spain. [3]Department of Genetics, Microbiology and Statistics, University of Barcelona, 08028 Barcelona, Spain. [4]University of Bordeaux, Inserm, Bordeaux Population Health Research Center, UMR 1219, F-33000 Bordeaux, France. [5]Université Clermont Auvergne, INRAE, UNH, F-63000 Clermont Ferrand, France. [6]Department of Basic and Clinical Neuroscience, Maurice Wohl Clinical Neuroscience Institute, Institute of Psychiatry, Psychology and Neuroscience, King's College London, London SE5 9NU, UK. [7]Brain Plasticity Group, Swammerdam Institute for Life Sciences, Center for Neuroscience, University of Amsterdam, 1098 XH Amsterdam, The Netherlands. [8]Institute of Molecular Regenerative Medicine, Spinal Cord Injury and Tissue Regeneration Center Salzburg, Paracelsus Medical University, Salzburg 5020, Austria. [9]Pharmacology Section, Department of Pharmacology, Toxicology and Medicinal Chemistry, Faculty of Pharmacy and Food Sciences, and Institut de Neurociències, University of Barcelona, 08028 Barcelona, Spain.

[*]Corresponding authors: Dr. Raúl González-Domínguez, Faculty of Pharmacy and Food Sciences, University of Barcelona, Av. de Joan XXIII, 27-31, 08028 Barcelona (Spain), Tel. +34-934024513, E-mail: raul.gonzalez@ub.edu; Prof. Cristina Andres-Lacueva, Faculty of Pharmacy and Food Sciences, University of Barcelona, Av. de Joan XXIII, 27-31, 08028 Barcelona (Spain), Tel. +34-934034840, E-mail: candres@ub.edu

Table S1. Concentrations (expressed as mean ± standard deviation, µg L^{-1}) detected within the cases and controls subjects for the metabolites identified to be associated with cognitive decline by LASSO regression in the discovery (Bordeaux) and validation (Dijon) sample sets.

**Metabolites**	**Bordeaux sample set**		**Dijon sample set**	
	**cases**	**controls**	**cases**	**controls**
Phenolic acids and derivatives				
Vanillin	21.3 ± 38.2	29.3 ± 45.8	7.2 ± 31.1	13.2 ± 47.7
3-Hydroxybenzoic acid sulfate (3-HBA-S)	8.2 ± 13.6	8.6 ± 12.1	14.5 ± 19.0	16.1 ± 18.0
2-Hydroxybenzoic acid (2-HBA)	189.2 ± 1508.2	100.5 ± 405.6	162.1 ± 1200.9	50.1 ± 185.7
3,4-Dihydroxybenzoic acid (3,4-DHBA)	40.8 ± 93.9	45.3 ± 65.2	32.4 ± 103.6	23.6 ± 26.8
3-Hydroxyphenylacetic acid sulfate (3-HPAA-S)	52358.7 ± 104446.7	52881.8 ± 121984.6	60125.6 ± 132259.5	23424.6 ± 71751.2
Dihydroferulic acid sulfate (DHFA-S)	3.0 ± 8.7	4.0 ± 8.9	2.7 ± 5.9	2.7 ± 6.1
3-Hydroxyhippuric acid (3-HHA)	49.1 ± 58.5	50.9 ± 66.4	33.8 ± 41.4	42.2 ± 50.5
Xanthine alkaloids				
3-Methylxanthine	51.0 ± 140.8	75.5 ± 168.1	80.6 ± 173.0	65.4 ± 113.3
Caffeine	1202.7 ± 1403.1	888.0 ± 1044.1	692.6 ± 943.4	687.5 ± 854.2
1,3-Dimethyluric acid	46.9 ± 34.7	40.5 ± 29.8	34.9 ± 38.2	40.7 ± 33.5
Artificial sweeteners				
Saccharin	11.5 ± 35.4	6.8 ± 26.4	5.9 ± 25.2	3.6 ± 13.8
Acesulfame K	7.6 ± 35.3	5.2 ± 24.5	2.8 ± 9.3	5.6 ±

				29.2
Other food-related metabolites				
Umbelliferone sulfate	1.7 ± 1.6	3.4 ± 13.9	1.3 ± 2.2	1.6 ± 3.7
2-Furoylglycine	9.6 ± 30.0	11.4 ± 41.4	6.8 ± 20.2	5.0 ± 9.5
Tartaric acid	111.4 ± 140.6	97.1 ± 134.4	101.5 ± 193.5	74.7 ± 142.2
Proline betaine	278.3 ± 256.5	220.2 ± 222.0	277.6 ± 292.7	315.8 ± 275.2
Enterolactone sulfate	1.1 ± 1.6	1.4 ± 1.7	0.2 ± 1.0	0.1 ± 0.5
5-(4'-Hydroxy-3'-methoxyphenyl)-γ-valerolactone sulfate (MHPV-S)	21.4 ± 55.5	34.2 ± 74.5	35.0 ± 101.3	19.0 ± 55.6
Ergothioneine	1268.3 ± 1637.0	1308.8 ± 1807.0	272.3 ± 290.7	381.7 ± 398.5
Ethyl sulfate	15.8 ± 44.6	13.8 ± 44.1	7.6 ± 19.9	4.6 ± 14.9
cis-Resveratrol 3-sulfate	6.8 ± 8.2	5.7 ± 6.4	20.6 ± 30.4	22.3 ± 34.3
Aromatic amino acid derivatives				
Indoxyl sulfate	1205.8 ± 809.7	1291.9 ± 690.6	745.1 ± 410.3	706.1 ± 388.9
4-Hydroxyphenylacetic acid glucuronide (4-HPAA-G)	82.8 ± 281.2	46.9 ± 180.5	76.0 ± 324.2	21.3 ± 114.8
3,4-Dihydroxyphenylacetic acid sulfate (DOPAC-S)	9019.7 ± 21384.7	9205.1 ± 26811.2	12516.7 ± 29668.5	5275.2 ± 11550.2
4-Hydroxyphenyllactic acid sulfate (4-HPLA-S)	452.1 ± 1055.8	440.1 ± 1239.4	499.6 ± 1305.7	212.2 ± 629.3
5-Hydroxytryptophan	93.0 ± 48.9	93.4 ± 43.4	154.2 ± 115.2	204.0 ± 181.2
4-Methylcatechol sulfate (4-MeCAT-S)	3906.2 ± 4286.8	5058.7 ± 8976.6	4963.8 ± 7482.6	3888.8 ± 4501.6
Phenylacetylglutamine	1025.6 ± 726.6	857.4 ± 536.8	1050.2 ± 611.4	951.3 ± 647.0

p-Cresol sulfate	4395.1 ± 1980.6	4224.9 ± 1894.8	3773.7 ± 1883.7	3282.5 ± 1815.3
Serotonin	77.0 ± 29.9	76.5 ± 27.0	47.0 ± 38.5	54.8 ± 41.9
Fatty acids and derivatives				
Myristic acid	17318.1 ± 11356.4	15060.6 ± 10631.2	20858.1 ± 7616.1	18396.0 ± 5991.8
Lauric acid	2989.4 ± 2704.4	2592.3 ± 2172.4	11949.8 ± 7133.1	10055.2 ± 5493.6
Linoleoyl-carnitine	89.1 ± 39.5	76.0 ± 34.1	53.8 ± 5.5	54.6 ± 10.9
Other endogenous metabolites				
Betaine	25364.7 ± 11863.7	29467.6 ± 12956.8	8044.8 ± 4121.9	8149.0 ± 3630.6
Dehydroepiandrosterone sulfate (DHEAS)	593.8 ± 502.3	666.3 ± 527.8	370.8 ± 309.1	356.1 ± 292.0
Citric acid	5608.7 ± 5315.1	6596.5 ± 6718.9	3800.3 ± 3739.1	3970.9 ± 3324.0
Thiamine	26.2 ± 37.5	26.2 ± 28.7	6.9 ± 19.5	7.7 ± 16.6

Table S2. Metabolites associated with subsequent cognitive decline in the total sample (i.e., combination of the populations from Bordeaux and Dijon) identified by least absolute shrinkage and selection operator (LASSO) penalized conditional logistic regression.

**Metabolites**	**Odds ratio**
Phenolic acids and derivatives	
3-Hydroxybenzoic acid sulfate (3-HBA-S)	0.87

Hippuric acid (HA)	0.98
Vanillin	0.83
Xanthine alkaloids	
3-Methylxanthine	0.86
1-Methylxanthine	0.96
Artificial sweeteners	
Saccharin	1.26
Other food-related metabolites	
Umbelliferone sulfate	0.91
Ergothioneine	0.90
Aromatic amino acid derivatives	
Phenylacetylglutamine	1.23
p-Cresol glucuronide	1.19
5-Hydroxytryptophan	0.89
5-Hydroxyindoleacetic acid	1.10
4-Hydroxyphenyllactic acid sulfate (4-HPLA-S)	1.12
Fatty acids and derivatives	
Oleic acid	1.12
Linoleic acid	1.19
Lauroyl-carnitine	1.18
Oleoyl-carnitine	1.18
Other endogenous metabolites	
Alanine	1.09
Glutamic acid	1.14
Carnosine	1.22
Creatinine	1.00
Guanosine	1.06
Riboflavin	1.02
Pantothenic acid	1.13

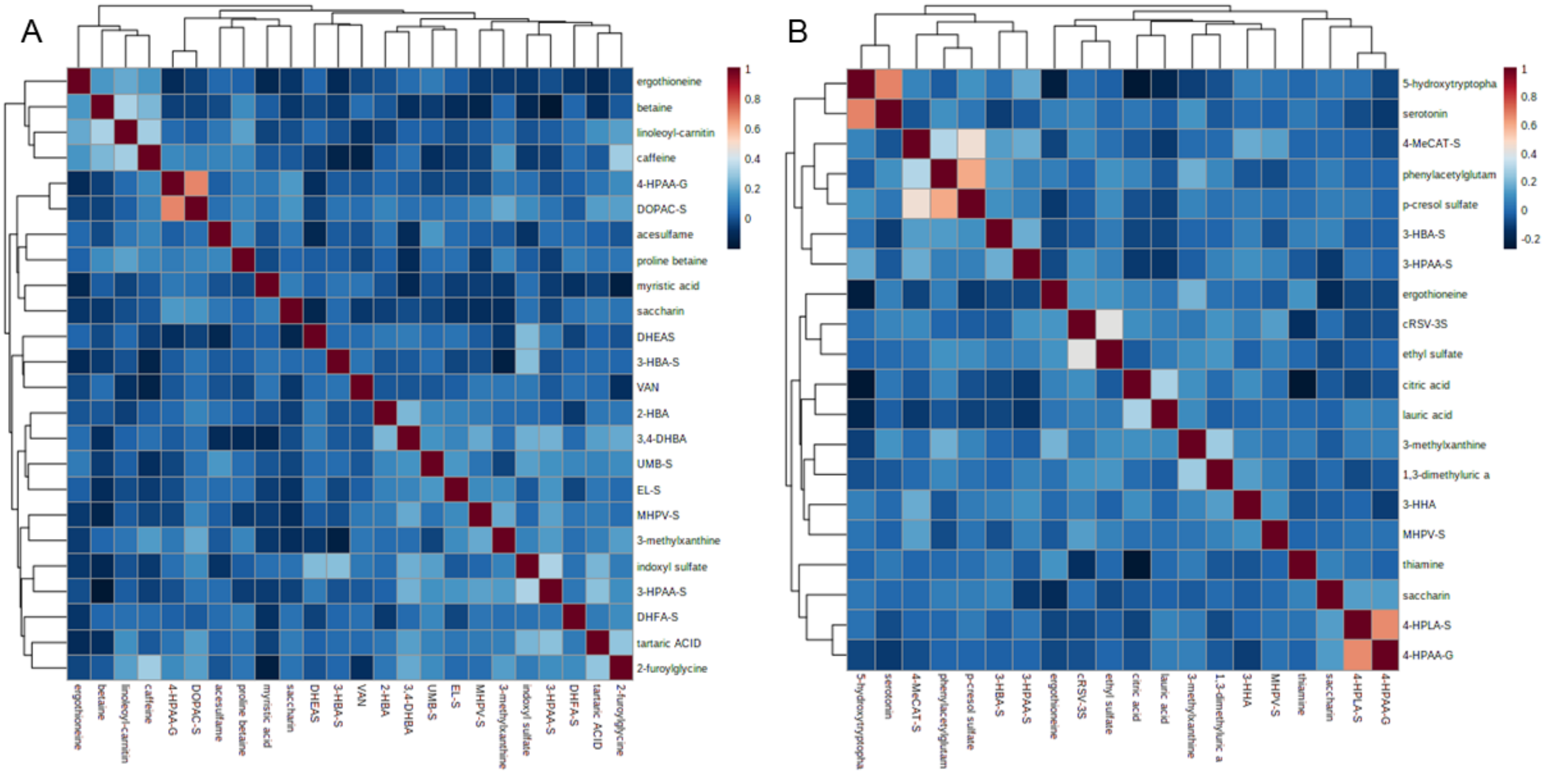

Figure S1. Pearson's correlation analysis between the metabolites that were identified by bootstrap-enhanced least absolute shrinkage and selection operator (LASSO) penalized conditional logistic regression to be associated with subsequent cognitive decline in the discovery (Bordeaux, A) and validation (Dijon, B) sample sets.

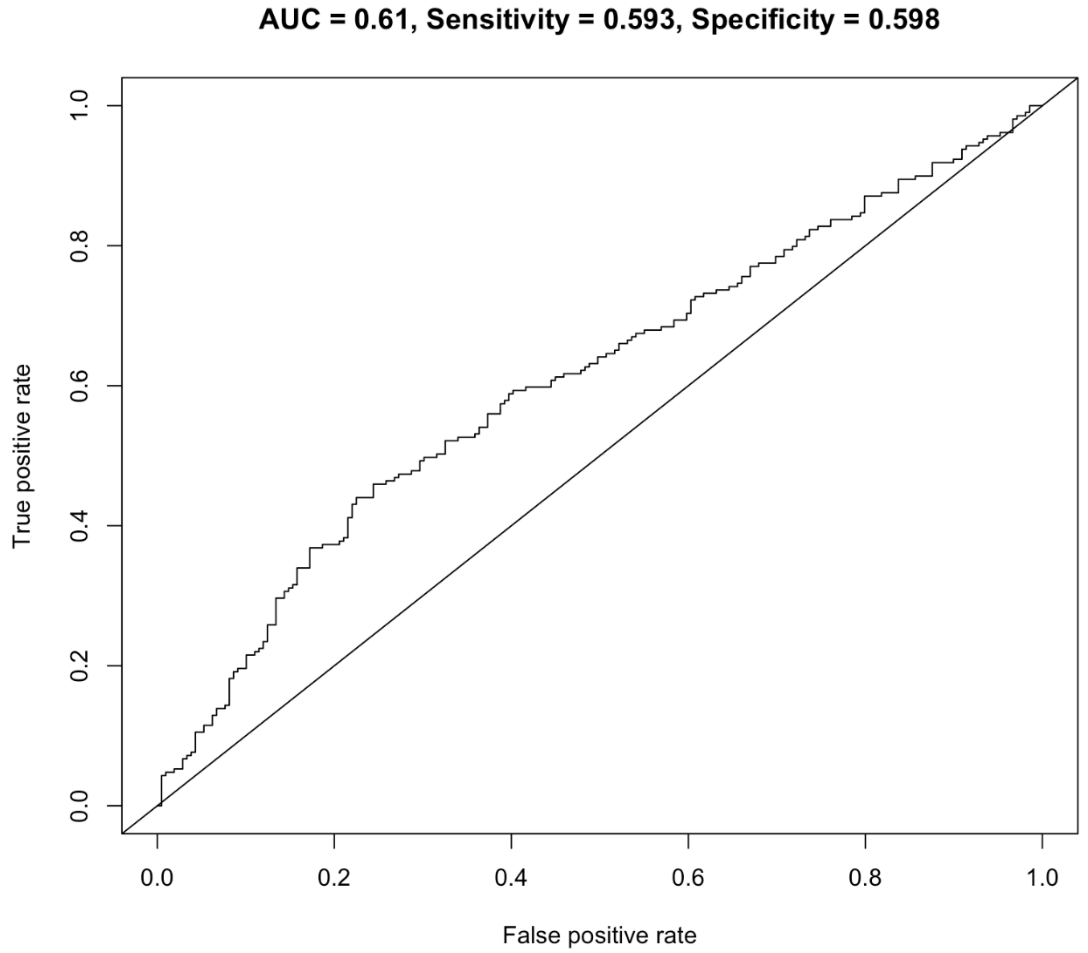

Figure S2. Receiver operating characteristic curve evaluating the performance of the 24-metabolite set identified in the discovery phase to predict the odds of CD in the external validation set.
